# External validation study of a clinical decision aid to reduce unnecessary antibiotic prescriptions in women with acute cystitis

**DOI:** 10.1186/s12875-017-0660-y

**Published:** 2017-10-02

**Authors:** Warren J. McIsaac, Rahim Moineddin, Ildikó Gágyor, Tony Mazzulli

**Affiliations:** 1 Granovsky-Gluskin Family Medicine Centre, Ray D. Wolfe Department of Family Medicine, Sinai Health System, Mount Sinai Hospital, 60 Murray Street, Toronto, M5T 3L9 Canada; 2 Department of Family and Community Medicine, University of Toronto, Toronto, Canada; 3 Department of General Practice, Göttingen University Medical Centre, Göttingen, Germany; 4 Department of Microbiology, Mount Sinai Hospital and University Health Network, Toronto, Canada; 5 Department of Laboratory Medicine and Pathobiology, University of Toronto, Toronto, Canada

**Keywords:** Antibiotic prescriptions, Decision aid, Cystitis

## Abstract

**Background:**

Empirical prescribing of antibiotics to women with symptoms of acute cystitis prior to culture results is common, but subsequent culture results are often negative. A clinical decision aid for prescribing decisions in acute cystitis was previously developed that could reduce these unnecessary antibiotic prescriptions but has not been validated. This study sought to validate this decision aid for empirical antibiotic prescribing decisions in a new cohort of women with suspected acute cystitis.

**Methods:**

External validation study of a clinical decision aid in 397 women with symptoms of acute cystitis, involving 230 Canadian family practitioners across Canada between 2009 and 2011. The sensitivity and specificity of the decision aid compared to a gold standard positive urine culture (defined as ≥10^2^ cfu/ml (≥10^5^ CFU/L)) was determined, and compared with physician management, and the earlier development cohort study estimates. Other outcomes assessed were total antibiotic prescriptions, unnecessary antibiotics for negative urine cultures, and recommendations for urine culture testing. Chi-square tests were used for unpaired comparisons, adjusted for physician clustering. McNemar’s test was used for paired comparisons.

**Results:**

There were 245/397 (61.7%) positive urine cultures. The cystitis aid sensitivity was 202/245 (82.5%, 95% Confidence Interval (CI)) = 77.1%, 86.8%), compared to 167/208 (80.3%) in the previous development cohort (*p* = 0.54), and 239/245 (97.6%) by family physicians in the current study (*p* < 0.001). Specificity was low for physicians (10/152, 6.6%) compared to the decision aid (54/152, 35.5%; *p* < 0.001, resulting in more antibiotic prescriptions by physicians (381/397, 96.0%) than would occur with decision aid recommendations (300/397, 75.6%, *p* < 0.001). Unnecessary antibiotic prescriptions where urine cultures were negative would be reduced an absolute 11.1% with cystitis aid recommendations (98/397, 24.7%) compared to usual physician care (142/397, 35.8%; *p* = 0.001). Urine cultures would also be reduced (97/397, 24.4% decision aid vs 351/397, 88.4% physicians; *p* < 0.001).

**Conclusions:**

A 3-item clinical decision aid demonstrated reproducible accuracy in two cohorts of women with acute cystitis symptoms. Clinically important reductions in total and unnecessary antibiotic use, as well as urine culture testing, could result with routine clinical use compared to current empirical physician management practices.

**Electronic supplementary material:**

The online version of this article (10.1186/s12875-017-0660-y) contains supplementary material, which is available to authorized users.

## Background

Uncomplicated urinary tract infection (UTI) is one of the most common bacterial infections encountered in the community [1]. Most are cases of acute cystitis in women where the causative organism is *Escherichia coli* in 75 to 95% of instances [1]. Over time, the prevalence of antibiotic resistant *E.coli* has increased [2]. The volume of antibiotics prescribed in the community is known to be associated with resistance at both a national and individual level [3, 4]. Reducing unnecessary antibiotic use in primary care can reduce community resistance levels [5]. However, determining whether antibiotics are needed can sometimes be challenging.

In acute cystitis, women present with uncomfortable symptoms whereas a urine culture result that could identify if a uropathogenic bacteria is present may take up to 3 days. Physicians typically prescribe antibiotics empirically based on clinical judgement [6], an approach endorsed in expert guidelines [1]. However, in 40% of empirically prescribed antibiotics for acute cystitis, urine cultures are negative or there is no significant growth [7].

Previously, a clinical decision aid was developed that demonstrated the potential to reduce total and unnecessary antibiotic use in managing acute cystitis compared to usual family physician care [7]. However, an attempt to validate the aid in community practice found one of the variables was not reliable, necessitating a modification to the original aid [8]. The modified acute cystitis decision aid criteria are dysuria (burning or discomfort with urination), a positive urine for leukocytes (>trace amount), and urinary nitrites (any present) [8]. If two or more criteria are present, a > 70% prevalence of a positive culture was demonstrated [8] and empirical antibiotics are suggested, consistent with expert recommendations [1, 6]. With one or fewer criteria (26–38% positive cultures) [7, 8], a culture is recommended with antibiotic treatment based on culture results.

Standards for validating clinical decision aids require independent validation in different populations to ensure reproducibility prior to clinical application [9, 10]. The objective of this study was to assess the validity of this modified acute cystitis decision aid by determining its performance in a new cohort of women who visited family physicians with cystitis symptoms. Specifically, we sought to determine the sensitivity and specificity of the cystitis decision aid for identifying bacterial infection, the reproducibility of these estimates compared with the development cohort estimates [8], and the potential impact of the decision aid on antibiotic use compared with usual clinical care by physicians.

## Methods

### Study design and setting

A pragmatic, prospective observational study of women presenting to family physicians with symptoms of cystitis was conducted. Pragmatic studies seek to include typical patients encountered in routine clinical care settings and managed with usual expertise and resources [11]. The TRIPOD statement (Transparent Reporting of a multivariable prediction model for Individual Prognosis or Diagnosis) format for reporting prediction model validation study results was followed [10].

Between 2009 and 2011, 15,742 family and general practitioners from across Canada were randomly selected (2008 Scott’s Canadian Medical Directory) and contacted about a study of acute cystitis. Sampling was stratified by province, proportional to the national distribution of physicians. Physicians were eligible if they conducted community family or general practice, provided care to adult women, and did not limit their practice to psychotherapy, emergency medicine, palliative care, sports medicine, or hospitalist care. Materials were available in English and French.

### Study participants and data collection

Participating physicians were asked to assess women presenting with new urinary tract symptoms where they considered acute cystitis a possible diagnosis. Physicians prospectively enrolled up to four women at their discretion, according to specified inclusion and exclusion criteria. Females 16 years of age or older presenting with new urinary symptoms suggestive of uncomplicated cystitis were eligible. Excluded were children, men, pregnant women, nursing home residents and those unable to understand English or French.

Standardized forms to document clinical findings and urine chemical strips (Siemens Multistix 8 SG, Siemens Healthcare Diagnostics Inc., USA), to test for leukocyte esterase and nitrites, were provided. A urine culture was requested for all women. Test strips and urine cultures physicians indicated they would not normally have ordered were paid for from study funds. No company or funding body had any role in the design, collection or analysis of the data, or drafting of the manuscript. Urine cultures were submitted to community laboratories that physicians’ routinely utilized. Testing procedures were not standardized in order to reflect regular clinical practice.

Physicians documented selected co-morbidities and whether an antibiotic was prescribed. The cystitis decision aid criteria were not provided and were not identifiable on the data collection form. As cultures were requested in all cases, physicians recorded whether they would have normally performed a dip urinalysis or ordered a urine culture, to reflect their usual practice. Women provided consent separately for the urine culture result.

The reference standard for diagnosis of a urine infection was a positive urine culture with a colony count ≥10^2^ cfu/ml (10^5^ CFU/L), as recommended for symptomatic women [12]. Each culture report was reviewed by two investigators, one of whom was a microbiologist (TM), to determine if an infection with a significant uropathogen was present. The laboratory culture reports were reviewed blinded to the clinical variables that family physicians had recorded on a separate form.

### Outcomes and statistical analysis

The primary outcome to assess the cystitis decision aid performance was the accuracy for identifying infection, indicated by the sensitivity and specificity of the aid. These estimates were compared to those from the original development cohort study to assess reproducibility [8]. The proportion of unnecessary urine cultures, total antibiotics and unnecessary antibiotics recommended were also compared with development cohort estimates [8], and for usual clinical care as recorded by physicians in the current study, to assess potential clinical impact.

Sensitivity of the decision aid was determined as the proportion of all positive urine cultures that would be identified in women with two or more criteria (antibiotic recommended), and specificity as the proportion of negative cultures in those with 1 or fewer criteria (culture recommended). Physician sensitivity and specificity was estimated similarly using their recommendation for an antibiotic. An antibiotic prescription was considered unnecessary if the subsequent urine culture result was negative.

The sample size was based on estimating resistance rates for a separate report using this data [13]. All women from that study with clinical information needed to apply the decision aid, and a culture result, were eligible for the current validation study. Categorical variables were described using frequencies and percentages, with means and ranges for continuous variables. The sensitivity and specificity of the decision aid threshold of 2 or more criteria present was determined in relation to the gold standard positive urine culture result. These estimates were compared to development cohort estimates [8] using a Pearson’s chi-square test, adjusted for the clustering of cases by physician (STATA version 12; Statacorp, Texas). McNemar’s test for paired comparisons was used to compare the proportion of urine cultures, unnecessary cultures, total antibiotics prescribed and unnecessary antibiotics recommended by the decision aid to observed care by physicians. Complete–case analysis was used to handle missing data [10]. Women not included because of missing culture results were compared with women included in the analysis for differences in predictor or outcome variables. The Research Ethics Board of the Mount Sinai Hospital (Toronto, Ontario) approved the study.

## Results

**Fig. 1:**
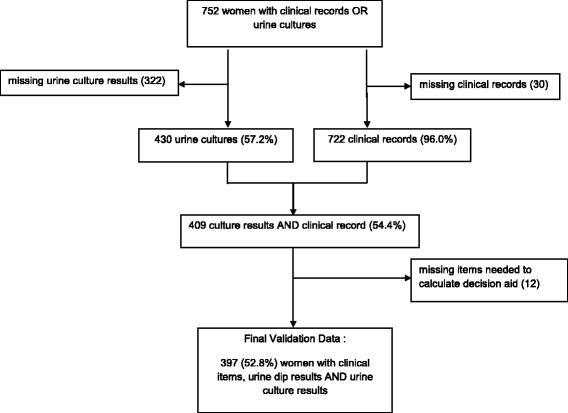
Final sample of women with acute cystitis, clinical information, urine dip test and urine culture results

In total, 330 family and general practitioners assessed 752 women from across Canada. There were 722 (96.0%) with complete clinical records and 430 (57.2%) were urine culture results were obtained. The final validation cohort for this analysis included 397 (52.8%) women assessed by 230 (69.7%) physicians with culture results, clinical information and dip urinalysis results needed to calculate the decision aid (Fig. 1).

Women with clinical records (*n* = 722) excluded from the validation cohort analysis (*n* = 325) due to missing urine cultures, dip urinalysis or clinical information needed to calculate the decision aid data did not differ from included women in the proportion with dysuria or urine nitrites, but were less likely to have positive leukocytes (Additional file 1: Table S1). Otherwise, excluded women were similar in clinical characteristics and management by physicians. Excluded women were younger than those included in the validation cohort analysis, but similar in age to women in the development cohort. No association was found between age and having a positive urine culture in either the validation (*p* = 0.39) or development cohorts (*p* = 0.69).

All provinces in Canada were represented (Table 1). Women ranged in age from 16 to 93 (mean 50.8 years), and 51.1% were older than 50 years of age. Cases were typical of acute uncomplicated cystitis with 363 (91.4%) women having at least two symptoms of dysuria, frequency or urgency, and 246 (62.0%) had all 3. Fever was infrequent (3.3%). While flank discomfort was reported by 69 (17.4%) women, there was only one diagnosis of pyelonephritis (0.3%). The prevalence of positive urine cultures was 245/397 (61.7%, 95% Confidence Interval (CI) = 56.6%, 66.6%). Physicians would have ordered a urine culture in 351(88.4%, 95% CI = 83.9%, 91.8%) presentations and prescribed an antibiotic empirically for 381 (96.0%, 95% CI =93.4%, 97.6%), with 19(4.8%) indicated as delayed prescriptions.

**Table 1:** Characteristics of women with clinically suspected acute uncomplicated cystitis: External validation cohort and development cohort [8]

	Validation Cohort		Development Cohort	
Characteristic	Number	Percent	Number	Percent
Total	397	100.0	331	100.0
Age				
≤ 50 years old	194	48.9	211	63.8
> 50 years of age	203	51.1	120	36.3*
Province				
British Columbia	61	15.4	56	16.9
Alberta	44	11.0	25	7.6
Saskatchewan	8	2.0	13	3.9
Manitoba	8	2.0	11	3.3
Ontario	208	52.4	195	58.9
Quebec	27	6.8	3	0.9
New Brunswick	9	2.3	8	2.4
Nova Scotia	9	2.3	12	3.6
Prince Edward Island	7	1.8	2	0.6
Newfoundland	16	4.0	6	1.8
Symptoms				
Dysuria	335	84.4	262	79.2
Frequency	349	87.9	304	92.1
Urgency	315	79.4	273	83.2
History of fever	13	3.3	13	4.0
Flank pain	69	17.4	83	25.4*
Management				
Would order culture	351	88.4	259	78.7*
Prescribed antibiotic	381	96.0	292	88.5*
Urine leukocytes (>trace)	316	79.6	243	73.4
Urine nitrites (any)	123	31.0	88	26.6
Positive Urine Culture	245	61.7	208	62.8

*p < 0.01, adjusted for clustering by physician

Compared to the development cohort [8] (Table 1), there were no differences in the distributions of decision aid predictor variables (dysuria, leukocytes, nitrites). The development cohort was younger (45.2 years) than the validation cohort (50.8 year, *p* < 0.001). More culture results were obtained for the development cohort (84.6%) than the validation cohort (52.8%, *p* < 0.001). However, the prevalence of positive cultures in each cohort was not different (62.8 vs 61.7%, *p* = 0.76). Physicians were more likely to prescribe an antibiotic and order a urine culture in the validation cohort (*p* < 0.01).

There was a direct association between the number of decision aid criteria and a positive urine culture (*p* = 0.0002, Table 2) in this validation cohort. For 300 (75.6%, 95% CI = 71.1%, 79.6%) women with 2 or more criteria, empirical antibiotics without obtaining a urine culture would be recommended, and 97 (24.4%, 95% CI = 20.4%, 29.0%) women would be advised to defer antibiotics pending urine culture test results (Table 2). The sensitivity of the decision aid treatment threshold for identifying women with a positive culture was 202/245 (82.5%, 95% CI = 77.1%, 86.8%) and the specificity was 54/152 (35.5%, 95% CI = 28.0%, 43.9%). An unnecessary antibiotic would be recommended to 98/397 women (24.7%, 95% CI = 20.5%, 29.5%) with a negative urine culture.

**Table 2:** The prevalence of positive urine cultures according to decision aid criteria and management recommendations

	Negative Cultures	Positive Cultures	Total
1. Number of Cystitis Decision Aid Criteria*			
0	12 (70.6%)	5 (29.4%)	17 (100%)
1	42 (52.5%)	38 (47.5%)	80 (100%)
2	76 (36.9%)	130 (63.1%)	206 (100%)
3	22 (23.4%)	72 (76.6%)	94 (100%)
Total	152 (38.3%)	245 (61.7%)	397 (100%)
2. Cystitis Decision Management Categories*			
No antibiotic, culture (0,1 criteria)	54 (55.7%)	43 (44.3%)	97 (100%)
Antibiotic (2,3 criteria)	98 (32.7%)	202 (67.3%)	300 (100%)
Total	152 (38.3%)	245 (61.7%)	397 (100%)

*p < 0.0002, Pearson chi-square test adjusted for physician clustering

### Cystitis decision aid performance in the validation and development cohorts (Table 3)

The decision aid sensitivity was not significantly different in the development (80.3%) and validation cohorts (82.5%, *p* = 0.54). However, specificity was lower in the validation cohort (35.5 vs 53.7% development cohort, *p* = 0.005). While the proportion of women recommended to undergo urine culture testing, start an antibiotic or prescribed an unnecessary antibiotic was statistically different between the two cohorts, the absolute difference was less than +/− 8% for each comparison.

**Table 3:** Comparison of acute cystitis decision aid performance in the development [8] and validation cohorts of women with acute cystitis

Outcome	Development Cohort [8] (2002)	Validation Cohort (2009–2011)	Absolute Difference	*p*-value*
Number of Family Physicians	205	230	–	–
Women with complete data	331	397	–	–
Prevalence of positive cultures	208/331 (62.8%)	245/397 (61.7%)	- 1.1%	0.76
1. Accuracy				
Sensitivity	167/208 (80.3%)	202/245 (82.5%)	+ 2.1%	0.54
Specificity	66/123 (53.7%)	54/152 (35.5%)	- 18.2%	0.005
2. Management				
Urine Cultures	107/331 (32.3%)	97/397 (24.4%)	- 7.9%	0.02
Antibiotics Prescribed	224/331 (67.7%)	300/397 (75.6%)	+ 7.9%	0.02
Unnecessary Antibiotics**	57/331 (17.2%)	98/397 (24.7%)	+ 7.5%	0.02

*adjusted for clustering by physician

**antibiotic prescription issued and subsequent urine culture was negative for bacteria

### Comparison of cystitis decision aid recommendations with observed physician care (Table 4)

Physician recommendations for antibiotics were more sensitive than the decision aid (97.6 vs 82.5%, *p* < 0.0001) in the validation cohort, but physician specificity was reduced by an absolute 28.9% (*p* < 0.001). This resulted in more unnecessary antibiotic recommendations with usual physician care (35.8%) than with decision aid recommendations (24.7%, *p* = 0.001), an absolute reduction of 11.1% and a relative reduction of 31.0% (95% CI = 22.6%, 38.5%) in unnecessary antibiotic prescriptions. The cystitis decision aid would recommend fewer urine cultures (64.0% absolute difference), total antibiotics (20.4%) and unnecessary antibiotics where urine cultures were negative (11.1%) compared to physician care (all *p* ≤ 0.001). This would correspond to 20 fewer antibiotic prescriptions for every 100 women assessed with the decision aid, and 11 fewer unnecessary antibiotics, compared to care by these family physicians.

**Table 4:** Comparison of physician management and acute cystitis decision aid recommendations management: validation cohort of women with acute cystitis, 2009–11

Outcome	Family Physicians	Cystitis Decision Aid	Absolute Difference (MD - Aid)	*p*-value*
1. Diagnosis				
Sensitivity	239/245 (97.6%)	202/245 (82.5%)	+ 15.1%	<0.0001
Specificity	10/152 (6.6%)	54/152 (35.5%)	- 28.9%	<0.0001
2. Management				
Urine Culture	351/397 (88.4%)	97/397 (24.4%)	+ 64.0%	<0.0001
Antibiotics	381/397 (96.0%)	300/397 (75.6%)	+ 20.4%	<0.0001
Unnecessary Antibiotics**	142/397 (35.8%)	98/397 (24.7%)	+ 11.1%	0.001

*McNemar’s test

**antibiotic prescription issued and subsequent urine culture was negative for bacteria

The decision aid accuracy and impact on antibiotic use compared to physician care in both cohorts is shown in Additional file 1: Table S2. The reduction in decision aid specificity in the two cohorts was also seen with usual physician management (22.1% development cohort vs 6.6% validation study (*p* < 0.001), and was lower in both cohorts for physician care than decision aid recommendations (53.3 and 35.5% respectively). Urine culture testing would also be reduced compared to physician care (*p* < 0.0001). Empirical antibiotics would have been reduced with decision aid recommendations by an absolute 20.8% (development cohort) and 20.4% (validation cohort) compared to physician management, and unnecessary antibiotics by 11.4 and 11.1%, respectively.

## Discussion

A clinical decision aid to inform empirical decisions about antibiotic treatment for women with suspected acute cystitis demonstrated reproducible accuracy and potential reductions in total and unnecessary antibiotic prescribing, and urine culture testing, in two national cohorts of women compared to observed care by family physicians.

The sensitivity of the decision aid for identifying women with culture positive acute cystitis for empirical antibiotic treatment was the same in two national, but different cohorts of women with cystitis symptoms, assessed 4 years apart. While family physicians were more sensitive with their prescribing decision in identifying women likely to have a positive urine culture, they demonstrated little specificity, resulting in almost all women receiving antibiotics. The specificity of the decision aid varied in the two cohorts, but this was also true for physicians, and the decision aid specificity was significantly higher than physician specificity in both cohorts. The superior specificity of the decision aid compared to usual empirical prescribing decisions translates into clinically important reductions in both total and unnecessary antibiotic prescriptions that would be realized in these women with typical cystitis presentations.

The impact of the cystitis decision aid on unnecessary antibiotic prescriptions was a clinically important 11% absolute reduction, and a 31% relative reduction. As physicians prescribe antibiotics empirically to most women with suspected cystitis, this corresponds to large reductions in total antibiotics and unnecessary antibiotic prescriptions. In a study from Wales, clinics that achieved large reductions in antibiotic prescribing over 7 years were able to reduce ampicillin and trimethoprim resistance in coliform urinary tract isolates [5]. Secondary benefits from the decision aid strategy are potential reductions in urine culture testing.

One question is whether women would be willing to wait for culture results before receiving antibiotics. This would occur in 24% of women assessed using the cystitis decision aid. A Dutch study found more than a third of women with cystitis were willing to delay antibiotic treatment [14]. In another randomized trial, no difference in symptom severity or duration was found with delayed antibiotics for cystitis compared to immediate treatment [15]. Ibuprofen has also been demonstrated to reduce symptom discomfort similar to antibiotics [16]. This could provide an practical alternative for women advised to wait for culture results.

A limitation of the current study was a large number of culture results that were not able to be obtained. In the development cohort, [8] 85% of culture results were obtained compared to 53% in the current study. This was due to a change in the method for obtaining consent in the validation study that relied on physicians giving women a consent form. Despite this, the prevalence of positive cultures in the two studies was the same. This is important as decision aid accuracy can be affected when disease prevalence varies in different populations [17]. As the prevalence of positive urine cultures and decision aid sensitivity in these two national cohorts of women with cystitis symptoms was the same, despite missing culture results, this suggests the decision aid is likely to apply in usual cystitis cases seen in primary care office settings. However, additional validation of the decision aid may be warranted given the missing cultures in this study. Validation in emergency departments or walk-in clinics would also be necessary to ensure the prevalence of culture-positive cystitis is similar, prior to use of the decision aid in those settings. Similarly, additional study of the performance of decision aid in developing countries would be needed prior to clinical use.

A second limitation was the recruitment of only 2% of physicians approached. Nonetheless, there were more than 230 different physicians involved from cities and towns across the entire country. In addition, the clinical characteristics of the women they assessed were typical of cases of acute cystitis that any family physician would see. Further, the similarity of the current results with those in the development study of a different cohort of women and family physicians provides additional reassurance that the decision aid is likely applicable in community family practice settings. The use of mid-stream urine samples as a reference standard could also be criticized. One study reported a poor correlation of mid-stream samples with catheter specimens, primarily for gram positive organisms [18]. However, for *E.coli*, which causes 75–95% of acute cystitis [1, 12] the positive predictive value was greater than 90% [18].

Different studies have used different microbiologic definitions for a positive culture. We chose a definition of ≥10^2^ cfu/ml (≥10^5^ CFU/L) as per North American standards [12]. Others have followed European microbiologic standards (> 10^3^ cfu/ml) [19, 20]. Nonetheless, the prevalence of culture positive UTI in the latter primary care studies (61% [19], 66% [20]) were comparable to the current study (62%). This may be because most women with established cystitis symptoms will have colony counts above both the ≥10^2^ and 10^3^ thresholds, resulting in little impact on the final prevalence of positive cultures. However, further assessments of the performance of the decision aid with different microbiologic definitions is advisable. One study reported some women with negative urine cultures improved when treated with antibiotics [21]. However, the definition of a positive culture in that study was a less sensitive microbiologic threshold of ≥10^5^ cfu/ml. It is possible some women deemed culture negative in that study may have been found to be culture positive using the lower 10^2^cfu/ml threshold used in the current study.

Other clinical decision aids have been proposed for acute cystitis [19, 20, 22]. Some involve complex scoring [19, 22], or have not undergone external validation in a new population to ensure reproducibility [19, 22]. One study compared a clinical symptom score and a urine dipstick score separately [20], whereas physicians utilize both clinical symptoms and urine dip stick results in deciding about a course of action. Symptoms combined with dipstick tests have been demonstrated to have better diagnostic accuracy than symptoms alone [23]. The study by Little et al. concluded that clinical and urine based scores had poor predictive accuracy, but did not compare their performance with that of usual physician care for diagnosis or antibiotic prescribing [20]. The current and previous studies [7, 8] found that cystitis decision aids have superior diagnostic performance compared to physicians, and the potential for clinically relevant reductions in unnecessary antibiotic use. Empirical antibiotic treatment by telephone without an office visit or urine culture has also been suggested as an appropriate strategy [6]. However, the impact of telephone management on antibiotic use in acute cystitis was not considered [6]. In a study of cystitis where symptoms and cultures were collected, 40% of women prescribed empirical antibiotics based on symptoms that could be assessed by telephone were associated with a negative urine cuture [7].

## Conclusions

This study provided external validation of a simple 3-item clinical decision aid for empirical antibiotic prescribing decisions in a new cohort of women with symptoms of acute cystitis, compared to a previous cohort used to develop the aid [8]. In addition, the potential of the aid to reduce antibiotic prescriptions and urine culture testing compared to usual care by family physicians was demonstrated. Given the frequency of cystitis and volume of antibiotics prescribed to manage this common condition, a randomized trial of the cystitis decision aid in practice seems warranted to further assess if this strategy can reduce unnecessary antibiotic utilization in primary care settings.

## Additional file

Additional file 1: Table S1. Comparison of women with suspected cystitis included and excluded from the validation cohort analysis; and with the development cohort [8]. **Table S2.** Comparison of physician management and acute cystitis decision aid recommendations in two cohorts of women with acute cystitis. (DOCX 17 kb)
