# Supplementary material for: External validation study of a clinical decision aid to reduce unnecessary antibiotic prescriptions in women with acute cystitis
Source: BMC Fam Pract. 2017 Oct 2;18:89. doi: 10.1186/s12875-017-0660-y (PMC5625594; doi:10.1186/s12875-017-0660-y)
Supplement: Additional file 1: Table S1. — Comparison of women with suspected cystitis included and excluded from the validation cohort analysis; and with the development cohort [8]. Table S2. Comparison of physician management and acute cystitis decision aid recommendations in two cohorts of women with acute cystitis. (DOCX 17 kb) [file 12875_2017_660_MOESM1_ESM.docx]

Additional file

Table S1 – Comparison of women with suspected cystitis included and excluded from the validation cohort analysis; and with the development cohort [8]

|  | **Validation Cohort (n=722) † †** | | **Development Cohort** | |
| --- | --- | --- | --- | --- |
| **Characteristic** | **Included in Analysis** | **Excluded from Analysis**** | |  |
| Total | 397 (100%) | 325 (100%) | | 331 (100%) |
| Age * |  |  | |  |
| ≤ 50 years old | 194 (48.9%) | 204 (64.4%) | | 211 (63.8%) |
| > 50 years old | 203 (51.1%) | 113 (35.7%) | | 120 (36.3%) |
| **Decision Aid Variables** |  |  | |  |
| Dysuria | 335 (84.4%) | 273 (84.0%) | | 262 (79.2%) |
| Urine leukocytes * | 316 (79.6%) | 214 (68.2%) | | 243 (73.4%) |
| Urine nitrites | 123 (31.0%) | 102 (33.8%) | | 88 (26.6%) |
| **Other Clinical Characteristics** | |  | |  |
| Frequency | 349 (87.9%) | 287 (88.3%) | | 304 (92.1%) |
| Urgency | 315 (79.4%) | 243 (74.8%) | | 273 (83.2%) |
| **Physician Management** |  |  | |  |
| Would order culture | 350 (88.2%) | 285 (87.7%) | | 259/329 (78.2%) |
| Prescribed antibiotic**†** | 381 (96.0%) | 305 (93.9%) | | 292/330 (88.5%) |
| **Positive Culture by Age**‡ |  |  | |  |
| < 50 years of age | 124/194 (63.9%) | - | | 131/211 (62.1%) |
| > 50 years of age | 121/203 (59.6%) | - | | 77/120 (64.2%) |

* *p*<0.01, included vs excluded, adjusted for physician clustering

**†** *p*=0.15, included vs excluded, adjusted for clustering

‡ *p*=0.39, included in validation cohort analysis; *p*=0.69 development cohort

** denominators for some characteristics < 325 due missing information

**††** women with clinical information to allow for comparison of the cohorts

Table S2 - Comparison of physician management and acute cystitis decision aid recommendations in two cohorts of women with acute cystitis

| **Outcome** | **Family Physicians** | **Cystitis Decision** **Aid** | **Absolute Difference** **(MD - Aid)** | ***p*-value†** |
| --- | --- | --- | --- | --- |
| **1.Diagnosis** |  |  |  |  |
| **Sensitivity** |  |  |  |  |
| development  cohort [8] | 197/208*  (94.7%) | 167/208  (80.3%) | + 14.4% | <0.0001 |
| validation  cohort | 239/245  (97.6%) | 202/245  (82.5%) | + 15.1% | <0.0001 |
| **Specificity** |  |  |  |  |
| development  cohort | 27/122*  (22.1%) | 65/122  (53.2%) | - 31.1% | <0.0001 |
| validation  cohort | 10/152  ( 6.6%) | 54/152  (35.5%) | - 28.9% | <0.0001 |
| **2. Management** |  |  |  |  |
| **Urine Culture** |  |  |  |  |
| development  cohort | 259/329* (78.7%) | 106/329  (32.2%) | + 46.5% | <0.0001 |
| validation  cohort | 351/397  (88.4%) | 97/397  (24.4%) | + 64.0% | <0.0001 |
| **Antibiotics** |  |  |  |  |
| development  cohort | 292/330*  (88.5%) | 224/330  (67.9%) | + 20.6%- | <0.0001 |
| validation  cohort | 381/397  (96.0%) | 300/397  (75.6%) | + 20.4% | <0.0001 |
| **Unnecessary**  **Antibiotic**** |  |  |  |  |
| development  cohort | 95/330*  (28.8%) | 57/330  (17.3%) | +11.5% | <0.0001 |
| validation  cohort | 142/397  (35.8%) | 98/397  (24.7%) | +11.1% | 0.0001 |
|  |  |  |  |  |

* Complete prescribing information for 330/331 women in the derivation cohort and for 329 regarding urine cultures

† McNemar’s test, matching data only (*n*=330 development cohort)

** antibiotic prescription issued and subsequent urine culture was negative for bacteria
